# Facilitators and obstacles in pre-hospital medical response to earthquakes: a qualitative study

**DOI:** 10.1186/1757-7241-19-30

**Published:** 2011-05-16

**Authors:** Ahmadreza Djalali, Hamidreza Khankeh, Gunnar Öhlén, Maaret Castrén, Lisa Kurland

**Affiliations:** 1 Department of Clinical Science and Education, Karolinska Institute, Södersjukhuset (KI SÖS), Stockholm, Sweden; 2 Nursing Department, University of Social Welfare and Rehabilitation, Tehran, Iran; 3 Department of Clinical Science, Intervention and Technology, Karolinska Institute, Stockholm, Sweden

## Abstract

**Background:**

Earthquakes are renowned as being amongst the most dangerous and destructive types of natural disasters. Iran, a developing country in Asia, is prone to earthquakes and is ranked as one of the most vulnerable countries in the world in this respect. The medical response in disasters is accompanied by managerial, logistic, technical, and medical challenges being also the case in the Bam earthquake in Iran. Our objective was to explore the medical response to the Bam earthquake with specific emphasis on pre-hospital medical management during the first days.

**Methods:**

The study was performed in 2008; an interview based qualitative study using content analysis. We conducted nineteen interviews with experts and managers responsible for responding to the Bam earthquake, including pre-hospital emergency medical services, the Red Crescent, and Universities of Medical Sciences. The selection of participants was determined by using a purposeful sampling method. Sample size was given by data saturation.

**Results:**

The pre-hospital medical service was divided into three categories; triage, emergency medical care and transportation, each category in turn was identified into facilitators and obstacles. The obstacles identified were absence of a structured disaster plan, absence of standardized medical teams, and shortage of resources. The army and skilled medical volunteers were identified as facilitators.

**Conclusions:**

The most compelling, and at the same time amenable obstacle, was the lack of a disaster management plan. It was evident that implementing a comprehensive plan would not only save lives but decrease suffering and enable an effective praxis of the available resources at pre-hospital and hospital levels.

## Background

Earthquakes are renowned as being amongst the most dangerous and destructive types of natural disasters known. More than one million earthquakes occur worldwide each year. Major earthquakes occur on average once every three years [1]. On a global scale a total of 400,000 people have been killed and 46 million affected by earthquakes and tsunamis, between 1991 and 2005 [2]. Consequently, an effective earthquake response is paramount in saving lives and limiting long term effects.

More than 90% of all the deaths caused by natural disasters occur in developing and underdeveloped countries [3]. Iran, a developing country in Asia, is prone to earthquake [4] and ranked as one of the most vulnerable countries in the world in respect to earthquakes and more than 180,000 people have died in earthquakes over the last 90 years [4-6].

**Figure 1:**
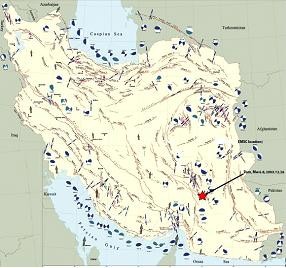
**The geographical place of the Bam earthquake**. Source: International Institute of Earthquake Engineering and Seismology, Iran

An earthquake with a magnitude of 6.7 on the Richter scale hit the city of Bam in Iran (Figure 1) [7]. The Bam earthquake is considered to be one of the 21st century's major earthquakes [8-10]. Approximately 40 thousand people perished and nearly 30,000 were injured [11,12].

Health services were rendered as non-functional (Table 1) [13]. More than 12 thousand injured people were evacuated, which put enormous demands on the disaster responding systems and admission sites [7,14].

**Table 1:** Damage of health care infrastructures due to the Bam earthquake

Health Facility	Number	% of Damage
Health house	95	100

Rural Health Center (RHC)	14	100

Urban Health Center (UHC)	10	100

Health posts (Urban)	5	100

Maternity facilities (as part of RHC)	5	100

Emam district hospital (public)	136 beds	50

Mahdieh maternity hospital (public)	54 beds	40

Aflatoonyan hospital (private)	65 beds	100

Emergency station (115)	1	100

Behvarz training center	1	100

District health network expansion center	1	100

District health care management center	1	100

Facualty of nursing and paramedics	(2000 sq.m.)	100

Dormitory of the faculty of nursing	(1500 sq.m.)	100

Source: World Health Organization (Ref: [7])

The medical response in disasters is normally accompanied by managerial, logistic, technical, and medical challenges [15-19] which was also the case in the Bam earthquake [12,20,21]. Our objective was to explore the medical response to the Bam earthquake with specific emphasis on the pre-hospital medical management during the first days.

Our aim was to identify obstacles and facilitators in pre-hospital medical response focusing on analyzing the organizational preparedness. We believe that the results can be used in designing an appropriate disaster management plan for both pre-hospital and the hospital services.

## Methods

The study was performed in 2008 on an interview based qualitative study using content analysis [22,23]. We used content analysis as a research method for the subjective interpretation of the content of interview data through a systematic classification process of coding and identifying concepts or patterns.

We conducted nineteen interviews with experts and managers of Iran's emergency and disaster medicine system. The participants were involved in the medical response to the Bam earthquake, including pre-hospital emergency medical services, the Red Crescent, and Universities of Medical Sciences. They had more than 5-year experience in disaster medicine and had participated in previous disasters.

The selection of participants was determined using a purposeful sampling method. The participants were included until saturation of each concept was reached and further data collection failed to contribute additional information. Sample size was given by data saturation.

Each interview lasted between 50 and 90 minutes. The interviews were conducted in Persian by the same interviewer, transcribed verbatim and then translated to English. Content analysis was performed on the data written in Persian, before translation.

The interview guide included a list of general questions used as a tool for initiating the interviews. Complementary probe questions were added when needed and, data collection and content analysis identified ideas, as is in accordance with the methodology.

During the open coding phase, all the interviews were read several times, and key words and phrases, incidents and facts in the text were noted. Primary codes were extracted. The codes and data were compared for similarities and differences.

Categories and sub-categories were developed. From the first interview, a preliminary set of codes, categories and sub-categories was created. These codes were described as the results [22-24]. In accordance with the methodology of content analysis [23,24]; this was performed by the same investigator for all interviews.

Data validation was performed through in-depth prolonged engagement with the data [22-24]. This procedure, combined with the available transcribed data and notes from the analysis process, are considered to ensure trustworthiness. Also, the transcriptions and a summary of primary result (codes and categories) checked by the participants in order to improve validity (member check).

### Ethical considerations

Ethical clearance of the study was obtained from the Natural Disaster Research Institute in Iran. Informed consent was obtained and all participants were informed that they could refuse to participate or withdraw from the study at any time.

## Results

### Demographic characteristics for the participants

The mean age of the participants was 43.5; well-educated in both health and medical sciences (Table 2).

**Table 2:** The background of the experts and managers participating in the current study

Age (years)	Mean (range)	43.5 (35-63)
**Gender (%)**	Male	100%

**Field of knowledge (n)**	Medical science	12
	Health management	4
	Emergency medicine	3

**Level of education (n)**	PhD	4
	General Practitioner	7
	Master of Science	5
	Bachelor of Science	3

n = number

### The process of triage, treatment and transportation of casualties

The pre-hospital medical services were divided into three categories; triage, treatment (emergency medical care) and transportation, and for each category in turn we had identified facilitators and obstacles (Table 3).

**Table 3:** Key factors related to the pre-hospital medical services during the medical response to the Bam earthquake

Pre-hospital medical response	*Obstacles*	*Facilitators*
**Triage**	- Absence of triage plan - Absence of triage teams - Absence of resources	- Medical specialists at the airport

**Treatment**	- Absence of disaster plan - Lack of Disaster Medical Assistance Teams - Shortage of resources	- Trained medical personnel from Army - Large number of medical volunteers

**Transportation**	- Absence of transportation plan - Lack of standardized transportation system - No control on transportation of the casualties	- Airlifting of casualties by Army

### Obstacles to Triage

The most important factor which affected the performance and workload of the medical services was the absence of triage. This was observed both at the pre-hospital and hospital level. The casualties were transferred to the airport or hospitals in nearby cities without being triaged. The lack of triage had detrimental consequences for both treatment and transportation, as well as the workload for the responding emergency medical services. For instance, a participant said *"Triage wasn't conducted during the medical response to the earthquake. Most of the casualties that were transported to nearby cities or to the airport only had minor injuries."*

The absence of a structured procedure and organized teams were the reasons for the lack of triage. In fact, there was no standardized operational plan for performing triage at the scene whatsoever. The lack of material resources was another contributing factor limiting the execution of triage. This included the lack of markers, tags, data forms, and basic medical equipment. Consequently, relatives and responders took the casualties directly to the airport or transferred them to hospitals at of nearby cities. A manager explained that *"With the lack of a disaster management plan and triage procedures, as well as incorrect policies, these were the main reasons that triage was not performed.*"

*"Triage was missed due to the lack of both triage teams and resources. There was no organized triage team on the scene. There was also a complete and apparent lack of essential triage resources during the first day.*"

### Facilitators of Triage

Groups of medical personnel, from the army and medically trained volunteers from the universities, were transferred to the earthquake area by the military air force within a few hours of locating the earthquake. Some of them were medical doctors, including surgeons and emergency medicine physicians. The airport was full of casualties needing medical attention and there was a continuous flow of earthquake victims being transported from the city to the airport. The medical personnel stayed at the airport and initiated medical treatment.

These specialists weren't planned to be part of the standardized emergency medical teams, and were not equipped with the necessary medical equipment or provided with resources to enable triage. However, they organized themselves as a response team and used the available resources and facilities to help the casualties at the airport as much as possible under the given circumstances. *"When we arrived in Bam, as individual medical officers and not part of a specific team, and without any pre-packed medical resources, we were struck by the large number of casualties at the airport." *reported by a medical professional.

Developing a triage system at the airport was the most important activity providing by this team. The airport was the only place where the earthquake victims were triaged, albeit in a limited fashion, without standardization or overall coordination and with a delayed start. They divided the airport waiting rooms into a few separate areas. They evaluated most of the casualties before further transport with airplanes and used the available resources for marking the casualties as triage groups. In addition, life saving medical care was administered. *"We organized the personnel as a team with the objective of conducting triage and giving life saving interventions on site at the airport. However, due to the shortage of resources, security and managerial problems, our system wasn't effective enough.*" an expert said.

Another participant reported that *"The absence of triage on scene made us perform primary triage at the airport. Performing triage decreased the overall workload for the medical service and transport organizations at all levels.*"

### Obstacles to Treatment

Emergency medical care on scene is life saving. Participants explain that this critical function was missed at the scene and basically all of the casualties were transferred to other cities without receiving initial medical attention. A medical doctor working at the airport said that *"All of the casualties arrived at the airport without first having medical attention before transportation from the earthquake site. Only, a few casualties had an intravenous line or wound dressings. In fact, the majority of the casualties received medical attention at the receiving hospitals.*"

However, the absence of a disaster management plan was the main reason for the lack of treatment. For instance, a manager said that *"We tried to provide on scene medical care for the casualties before taking them to the hospitals but it wasn't possible due to the lack of plan for how the medical system was supposed to manage the large number of casualties in a city structurally destroyed by the earthquake.*"

A disaster management plan along with emergency medical teams is the most important functions in order to guarantee appropriate medical care in a disaster area. During the Bam earthquake, a large number of disaster medical assistance teams were needed in order to provide emergency medical care. This need was not met. An expert explained that "*One of the main shortcomings in Iran's medical disaster management system, with respect to the Bam earthquake, was the lack of standardized disaster medical teams*."

Another expert mentioned that "*Due to the lack of structured medical teams, untrained medical volunteers that were involved in rescuing and caring for casualties. They were not organized as teams. They were like small islands and their performance was not good enough*. *Actually, we had to support them with medical and general equipment.*"

The interviewees emphasized the shortage of resources on the scene. Medical teams did not have enough equipment during the acute phase, and the destruction of all medical facilities made the situation worse. Consequently, medical services ceased during the first night. This, along with the cold weather, worsened the medical condition for the casualties.

A manager said that *"We had a considerable shortage of resources for providing medical services at the earthquake area during the first days."*

And another added that "*Working at the earthquake area amongst extensive destruction, a large number of casualties, with too few medical responders and with a lack of resources was difficult. As a result, medical services stopped during the first night. Besides, it was very cold and most of victims were exposed and could not keep warm. Consequently, some of them died due to exposure*."

### Facilitators of Treatment

Experienced and trained medical responders had enhanced the emergency medical response performance. Several organizations mobilized with the aim of reducing the impact of the Bam earthquake. Especially the army and the Red Crescent assisted the EMS. They sent medical teams to the scene, who participated in the search for buried victims, and contributed to the transportation of casualties. These teams included trained medical staff with experience from previous mass casualties, along with medical supplies. The army and the Red Crescent also provided logistics support. "*Military medical teams were one of the first teams that arrived at the earthquake site. They supported the pre-hospital medical system in every way, providing medical services, equipment and personnel.*"

"*Iran's Red Crescent sent many ambulances and medical teams to the earthquake area. They conducted rescue operations, provided basic medical care for the casualties, and transported them to the airport or nearby cities as required.*"

Many volunteers from the Universities of Medical Sciences arrived at the earthquake area in addition to the above mentioned organizations. Some of whom were well educated and had previous experience of disasters. Volunteers with advanced medical degrees could, partly, compensate for a lack of standardized medical teams.

The medical response could have been different had there been a disaster management plan, an organization for coordinating the rescue efforts and sufficient resource. "*The presence of medically trained volunteers helped the medical system to care for thousands of casualties on scene, despite them not being organized as standardized teams*" according to one of the interviewee.

Another study participant said that "*if the managers had organized the medical volunteers as coordinated teams, the medical services could have been conducted more effectively*."

Other responders were international emergency medical teams. These teams arrived late, when the earthquake site was cleared of trauma victims, hence despite having structured teams their contribution was insignificant.

### Obstacles to Transportation

The transportation of casualties to medical centres is an essential function in disaster response. The government decided to transfer all casualties to other cities since the Bam earthquake had destroyed all the local medical facilities. The lack of a disaster plan affected these operations also. In fact, there was no coordinated transportation plan at any level, neither was there an organization or a team responsible for the transportation of victims. At times, casualties were airlifted from the earthquake area without coordination with the receiving medical system. A participant mentioned "*There was no operational plan nor were there procedures for transportation. All decisions were made on the spur of the moment.*"

*"Casualties were, in some cases, transferred from the earthquake area to a specific city, and because of the inability to admit the casualties, they were referred to yet a second city*" according to another interviewee.

The lack of a coordinated plan for transportation of the casualties resulted in traffic chaos and a stop in air transportation. The roads were not controlled by the police and became blocked by vehicles. Additionally all transportation from Bam to the airport and the further evacuation by air was stopped during the first night because of darkness, very cold weather and lack of safety. A participant reported "*The roads were completely congested, to the extent that evacuating the casualties by road way was impossible.*"

"*Only a few hours after the arrival of the first response teams, the evacuation by air was stopped*" according to another participant.

Furthermore, there were no standardized transportation methods, neither for ground vehicles nor by air.

There were, also, no standard protocols for evacuation, a shortage of transportation vehicles and trained medical personnel. There was a long delay in initiating the evacuation which resulted in a disorganised evacuation of the earthquake casualties. This may have increased the mortality and the long term medical complications, e.g. spinal injury.

"*Since the arrival of rescue workers was delayed, some untrained response workers and laypeople began evacuating the casualties to nearby cities in private vehicles, without taking medical considerations.*"

Another expert said "*there weren't sufficient resources, equipment or ambulances. As a result, the casualties were evacuated without medical considerations."*

"*Victims transported by air must be done based on standardized protocols. Unfortunately many casualties were left on the floor of the airplanes without proper fixation or a plan for medical care during the flight." *as mentioned by another interviewee.

Furthermore, the absence of a prioritization for evacuation of the individual casualties was a problem. There were no rules or plans for the evacuation of casualties from the city to the airport, and from there on to the receiving cities. All casualties, both mild and severe, as well as relatives, were transported to the receiving cities, without a priority for the severe injured. This resulted in prolonged waiting times for all casualties involved. "*There was no control or security system at the airport."*

Another participant reported that "*medical priority was often missed while evacuating the casualties. Many casualties with mild or even without injuries were transported to other cities, while some casualties with severe injuries were still waiting for evacuation."*

### Facilitators of Transportation

The evacuation of thousands of victims from Bam in two days was one of largest rescue operations ever performed in the history of Iran. Ground transportation was the most common means of transporting victims on the first day and by air on the second. In fact, there were two evacuation waves. A small number of casualties were evacuated on the first day. The second wave started in the early morning on the second day and consisted of casualties evacuated mainly by air.

It was the air force's responsibility, along with the air transport organization, to provide the evacuation by air. In addition they carried managers, medical teams and equipment to the earthquake area.

A participant said *"The air force managed to reopen the airport, which had been damaged by the earthquake, and more than 10,000 casualties were evacuated within 24 hours through this airport."*

Another participant added that *"The air force and air transport organization concentrated all efforts on establishing a reliable evacuation path by air from Bam to the rest of the country."*

This situation was also seen in other cities, especially in the capital, Tehran. A manager quoted *"Several thousand casualties had been taken to Tehran's airport. The Air force established an air bridge between the airport and other large hospitals."*

## Discussion

In this article we focused on the pre-hospital issues of the early response phase to the Bam earthquake. The key elements in the pre-hospital medical response to disasters are correct triage, immediate treatment and in giving priorities for evacuation [25], which our study also addressed. Obstacles to the pre-hospital medical response could be summarized as the lack of a comprehensive disaster management plan, the absence of standardized disaster medical assistance teams, and a shortage of resources. While the facilitators were skilled medical volunteers and support from other organizations especially the army.

### Triage

During the medical response to a disaster, triage is needed at three locations: (1) on scene; (2) in the treatment and resuscitation area; and (3) at the receiving hospital [8].

The current study emphasized that the triage process was problematic, not performed on scene but was to a certain extent at the airport. All previous studies from the Bam earthquake [11,14,26], with exception of one [13], support the observation in the absence of triage.

Reports from other earthquakes show divergent results with respect to triage. During the tsunami in Thailand in 2004, field triage was performed and 70% of the victims were primarily treated in the field or triaged to emergency healthcare centres [27]. Yasin, however, explains that after the earthquake in Pakistan in 2005, most of the casualties received at the hospital were referred directly from the earthquake site without being triaged [28].

In summary, the lack of triage could affect medical response at all levels. In other disasters, triage has been performed to a varying extent. With the current medical response organization in Iran, hospitals should expect to receive many non-triaged casualties in the event of an earthquake. Therefore, some of the resources will be "unnecessarily" used for low priority cases, instead of the severely injured casualties. Triage is a vital issue in a disaster plan.

### Treatment

There was a lack of on site emergency medical treatment. This finding was supported by other studies [10,11,14,21]. Mirhashemi et al. studied 185 casualties of the Bam earthquake and saw that as much as 72.4-85.9% of the patients did not receive primary medical treatment on site [10].

Reports from other earthquakes show a similar situation worldwide, that medical response was inadequate on site, and most of the casualties were transported away from the earthquake area without initial medial treatment [28-30].

In summary, there was lack of immediate and adequate emergency medical care during the Bam earthquake. This could be one of the reasons for the high death toll in the Bam earthquake. Also, it is to be expected that the receiving hospitals not only receive non-triaged casualties but also casualties not having received primary medical treatment in the field. This lack of pre-hospital care will necessitate a large consumption of medical resources and strategic facilities to surge capacity. This point must also be considered in disaster planning.

### Transportation

This study shows that the overall effort of transporting victims was uncoordinated and insufficient. However, an air bridge did provide a means of rapid transportation for earthquake victims. Approximately 12,000 injured people were evacuated within 48-72 hours using civil and military aircrafts, road transportation and helicopters [31,32].

These findings are supported by other studies [10]. Furthermore, insufficient capacity of evacuation by air within the first 24 hours has been shown to lead to a higher mortality rate [10]. Consequently, this leads to patients being moved by non-standard methods [10].

Our observation that traffic jams inhibited transportation was confirmed by other researchers. Most of the roads leading both to and from Bam were blocked due to the rapid influx of rescue workers and by victims' relatives coming from neighbouring cities [14,33].

The effects of earthquakes on transportation are similar worldwide. For instance, on the first day after the Kobe earthquake in Japan in1995, only one person was transported by helicopter, and a total of 17 people were transported within the first 72 hours [1,34]. The family car was the most frequent means of transportation, and ambulances were used in only 26% of the transports [35]. Other studies have also reported similar observations [27,30,36].

We conclude that ground transportation is always affected in major earthquakes. In addition, air transportation services are often unavailable. The failure of transportation of casualties could seriously affect the medical response. Therefore, the receiving hospitals could receive the casualties only after a long delay. Thus, enabling these hospitals to prepare for incoming casualties, and effectuate their disaster management plan if it exists.

### Obstacles

#### Lack of disaster management plan

Timely and effective response to disasters requires an organized disaster response system which can provide the appropriate aid [10,37,38].

The lack of a disaster management plan was found to be the main obstacle in the pre-hospital medical management, with respect to triage, treatment, and transportation. Preparedness was not a priority for the Ministry of Health of Iran prior to the Bam earthquake, as explained by de Ville [18]. The major problem was the lack of coordination between the organizations responsible for disaster management, this in turn, is the consequence of the lack of both local and regional disaster management plans [12,14,26].

The lack of a disaster management plan has been recognized as a challenge worldwide regarding disaster response. The preparedness of the Ministry of Health in Pakistan was far from sufficient regarding the earthquake in 2005 [18]. Since there was no disaster management plan during the Gujarat earthquake, in India, 2001, most of the decision-making was completely ad hoc [36]. The problem with coordination in the response is also confirmed by Schwartz et al, as reported from the Tsunami in 2004 [27].

Our results emphasized the necessity in developing a comprehensive and integrated disaster management plan for the medical system.

#### Absence of disaster medical assistance team

Another obstacle in the pre-hospital medical response during the Bam earthquake was the absence of standardized disaster medical assistance teams (DMATs). DMATs defined as "mobile, trained medical teams that can be rapidly deployed during the acute phase of a disaster, provide medical treatment and relief activities, assisting in transferring casualties from disaster-affected areas to appropriate medical facilities" [34].

The absence of standardized DMATs effects triage, treatment and transportation. Although, no previous studies have clearly analyzed the role of DMATs in the Bam earthquake, two studies point out that physicians and responders had not received training for aid, rescue, and treatment in critical conditions [12,14].

There was a similar situation during the earthquakes in Taiwan (1999), and Japan (1995). Only a small number of medical teams were able to provide critical care needed during these earthquakes. These experiences actually lead to the development of disaster medical assistance teams and the recognition of their importance [30,34,39].

Taking all of this into account is of great importance in establishing DMATs in Iran.

#### Lack of resources

The post-earthquake environment has normally very limited medical resources in the face of overwhelming needs [40]. The shortage of medical resources during the early phase of the Bam earthquake was an important obstacle for pre-hospital medical management. Our study is one of few, which took the shortage of medical resources in the early phase into consideration. There was a noticeable shortage of beds, blankets, triage tags, medicine and intravenous fluids, during the first days after the earthquake [14,41].

Reports from other earthquakes show a similar situation worldwide. de Ville explained that in spite of an overwhelming budget, after the tsunami in 2004, the external responses were burdened by serious shortcomings [18]. Kohl et al. discusses also, the shortage of resources in the healthcare systems in the affected countries, before and during the Tsunami [42].

Limiting factors for the medical response during the Pakistan earthquake were operating space, equipment, supplies, and paramedical staff [28].

Based on these findings, it is essential to develop an effective logistics system, and define its role as part of a comprehensive disaster management plan in order to provide the necessary medical resources in the earthquake area.

### Facilitator

#### The military system

The current study illustrated the comprehensive role of military teams, especially their participation in the airlifting of casualties and the provision of necessary resources.

These results are supported by other studies [14,18,26,32,33] demonstrating the role of the military during the early phase of response. These among others; established field hospitals, triage, albeit to a limited extent, transporting medical and health care personnel to the disaster area and playing a significant role in air transportation of casualties to other receiving hospitals [14,18,26,32,33].

Similar experiences are reported in other disasters, e.g. the Tsunami in Thailand and Indonesia [27,43], the Chi-Chi earthquake in Taiwan [16,39] and the Gujarat earthquake in India [44].

As demonstrated, the army plays an important part in the early response to earthquakes. The army can be used in all parts of the medical response to an earthquake which should be taken into consideration when developing a comprehensive disaster management plan.

#### Skilled medical volunteers

Medical volunteers that came to the earthquake area, as part of a team or individually, during the early response to the earthquake were another facilitator. The first volunteer medical relief team consisted of 40 specialists, and arrived in the area a few hours after the event. These volunteers conducted the triage at the Bam airport [31]. Numerous teams consisting of physicians, paramedics, and volunteers from Universities of Medical Sciences from all over the country were dispatched to the area [33].

Volunteers played a crucial role in mass-casualty incidents [45]. Volunteer doctors from various backgrounds teamed up to meet the medical crisis in the Gujarat earthquake [36]. The rapid deployment of medical personnel from unaffected areas and volunteers from abroad helped key personnel handle the medical needs of the tsunami victims in Thailand [27]. This is also confirmed by Yasin et al. showing that medical teams composed of volunteers from Pakistan and abroad came in large numbers in response to the Pakistan earthquake [28].

In spite of the long delay, the role of international volunteer teams is important in helping the affected community. International organizations can assist the affected country by providing urgent medical services in proximity to the disaster area, thereby compensating for the shortages of medical facilities at national level [46].

The number of volunteers is virtually unlimited in Iran, and medical volunteers are most willing to assist [18]. Organizing the volunteers is not possible during the response phase of a disaster. However, it could be considered during the preparedness phase.

## Conclusions

This study was based on interviews from the managers at Iran's emergency and disaster medicine system, who were responsible for the initial response to the Bam earthquake. We focused on the initial triage, treatment and transportation. Skilled medical volunteers and the military medical teams were facilitators of the early phase of the medical response. The main obstacles to the pre-hospital medical response were the lack of a disaster management plan, the absence of disaster medical assistance teams and the overall lack of resources. The most compelling, and at the same time amenable obstacle, was the lack of a disaster management plan. It is evident that implementing a comprehensive plan would not only save lives but decrease suffering and enable an effective usage of the available resources. Due to the crucial role of pre-hospital care system in disasters there is a need for further investigation based on the result of this study to develop strategies for improving the system.

## List of Abbreviations

EMS: Emergency medical services; DMATs: Disaster medical assistance teams

## Competing interests

The authors declare that they have no competing interests.

## Authors' contributions

ARD was involved in the study conception and design, data collection, analysis, revision, editing and manuscript writing. HK was involved in the conception and design of study and took an active part in the data analysis and results interpretation. GO participated to the study conception and design, writing-up and finalization of the manuscript. MC contributed to analyze and interpret the data and to write the manuscript. LK participated to the study design, analysis and results interpretation and writing-up of the manuscript. All authors read and approved the final manuscript.
